# Secondary cutaneous marginal zone lymphoma arising in association with a Mohs micrographic surgery graft site

**DOI:** 10.1016/j.jdcr.2025.09.053

**Published:** 2025-11-03

**Authors:** Sarah J. Lange, Nicole Trepanowski, Robert E. LeBlanc, Jeremiah X. Karrs, Frederick Lansigan, Joi B. Carter

**Affiliations:** a Geisel School of Medicine at Dartmouth, Hanover, New Hampshire; b Department of Dermatology, Dartmouth-Hitchcock Medical Center, Lebanon, New Hampshire; c Department of Pathology, Dartmouth-Hitchcock Medical Center, Lebanon, New Hampshire; d Dartmouth Cancer Center, Dartmouth-Hitchcock Medical Center, Lebanon, New Hampshire

**Keywords:** basal cell carcinoma, BCC, cutaneous B-cell lymphoma, cutaneous marginal zone lymphoma, graft, marginal zone lymphoma, MMS, Mohs micrographic surgery, Mohs, MZL, skin graft

## Introduction

Marginal zone lymphoma (MZL) is a rare, low-grade B-cell malignancy. Though unknown, the pathogenesis has been linked to immune system dysregulation related to chronic inflammation, autoimmune disorders, and genetic abnormalities such as the t(14;18) (q32;q21) translocation that activates the NF-κB pathway.^1^ Cutaneous marginal zone lymphomas (CMZLs) are categorized as primary or secondary based on their origin. Primary cutaneous marginal zone lymphoma (pcMZL) represents 7% to 8% of all cutaneous lymphomas (25% of cutaneous B-cell lymphomas) and generally presents as localized skin lesions without extracutaneous involvement.^2^ In contrast, secondary cutaneous marginal zone lymphoma (scMZL) represents skin involvement by a marginal zone lymphoma of extracutaneous origin and is much less common, with Surveillance, Epidemiology, and End Results registry data from 1975-2016 identifying only 177 biopsy-confirmed scMZL cases compared to 1333 pcMZL cases.^3^

Chronic infections, autoimmunity, and immunodeficiency induce sustained activation of the lymphoid system, serving as a risk factor for lymphoma development.^4^ Here, we present a case of scMZL occurring at a skin graft site following Mohs micrographic surgery (MMS) for basal cell carcinoma (BCC) treatment, possibly incited by post-surgical inflammatory changes. Informed consent was obtained.

## Case report

**Fig 1:**
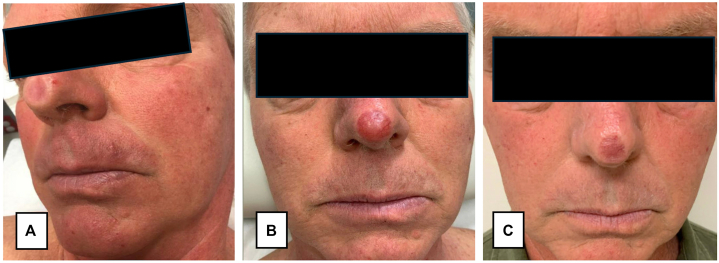
**A,** Well-healed graft site on the nasal tip 6 years post-Mohs micrographic surgical treatment of a basal cell carcinoma. **B,** Twelve years post-Mohs micrographic surgical treatment, the patient presents with telangiectasias and erythema with a boggy texture at the site of the previous nasal tip graft. **C,** Complete resolution of secondary cutaneous marginal zone lymphoma 2 months post-low dose radiation therapy.

A 70-year-old man presented with increased redness and swelling within a previous skin graft on the nasal tip, not noted at his skin exam 1 year prior. 12 years earlier, a BCC on the nasal tip was treated with MMS with a full-thickness skin graft repair from the right post-auricular area (Fig 1, *A*). Dermatologic history was significant for the previously mentioned BCC and a squamous cell carcinoma in situ on the left mandible treated with MMS with an intermediate linear closure. The patient was not immunosuppressed and had no other cancer history.

**Fig 2:**
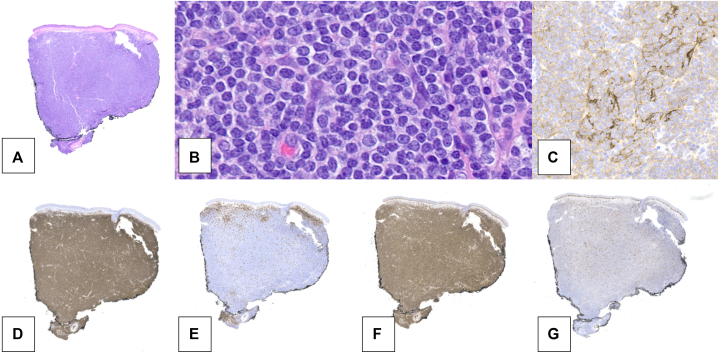
Punch biopsy, nasal tip, H&E, scanning **(A)** and high power **(B),** showing a dense lymphoid infiltrate filling the reticular dermis. Sheets of monotonous mature-appearing lymphocytes are punctuated by the occasional capillary (**B,** H&E, 400×). A rare tingible body macrophage is present, but no germinal center follicles are evident. CD21 immunohistochemistry reveals faint tufts of staining throughout the infiltrate suggesting germinal center follicles colonization (**C,** 200×). A vast preponderance of the infiltrate expresses CD20 (**D,** 10×). CD3 highlights spare T-lymphocytes (**E,** 10×). BCL2 is aberrantly expressed by B-cells (**F,** 10×). Ki-67 highlights scattered nuclei with no aggregates to indicate the presence of germinal center follicles (**G,** 10×).

Examination was notable for a 1 cm graft on the nasal tip with telangiectasias and increased erythema with a boggy texture (Fig 1, *B*). There were no signs of malignancy at the post-auricular site and no lymphadenopathy or splenomegaly. A punch biopsy of the nasal tip revealed a dense dermal infiltrate with sheets of monotonous B-cells co-expressing CD20 and BCL2 (Fig 2). CD3 highlighted sparse T-cells. CD21 showed small, moth-eaten dendritic cell networks suggesting the presence of colonized germinal center follicles. Lesional B-cells were negative for CD5, LEF1, Cyclin D1, Sox-11, and BCL-6. Kappa and lambda stain findings were insufficient to identify a light chain restriction, highlighting only scattered background cells. Clinical laboratory workup including complete blood count, comprehensive metabolic panel, lactate dehydrogenase, and serum protein electrophoresis (SPEP) was within normal limits, and infectious testing for Hepatitis B, Hepatitis C, and human immunodeficiency virus was negative. Peripheral blood flow cytometry identified a dominant circulating CD5-/CD10-kappa-restricted monoclonal B-cell population with a kappa to lambda ratio of 6.4. Computed tomography imaging of the chest, abdomen, and pelvis was unremarkable.

Ultimately, the patient was diagnosed with secondary cutaneous marginal zone B-cell lymphoma. He was treated with localized, low-dose radiation (4 Gray in 2 fractions of 2 Gray on 2 consecutive days), resulting in complete response (Fig 1, *C*). At 18 months after presentation, the patient developed recurrence at the prior graft site with histopathologic features consistent with his prior lymphoma, again demonstrating a dense B-cell infiltrate with kappa restriction and paucity of plasma cells. Positron emission tomography-computed tomography did not identify nodal or extranodal disease; however, bone marrow flow cytometry revealed a similar CD5-/CD10-kappa-restricted B-cell population, with a monoclonal IGH gene rearrangement matching the skin biopsy at presentation. The patient was started on a course of 4 weekly infusions of rituximab.

## Discussion

Herein, we present a case of scMZL associated with a MMS skin graft site. Differentiating pcMZL from scMZL is critical to guide therapeutic and prognostic discussions.^5^ In our case, the presence of circulating kappa-restricted B-cell population in blood and bone marrow concordant with the cutaneous infiltrate and identical clonality in skin and bone marrow biopsies support a diagnosis of scMZL over pcMZL. Normal SPEP and paucity of plasma cells on skin biopsy argue against monoclonal gammopathy of undetermined significance.

Histopathologic and immunohistochemic features of pcMZL and scMZL may overlap; pcMZL generally shows IgG class-switching with polymorphous infiltrates rich in T-lymphocytes, whereas scMZL more often demonstrates IgM predominance, sheet-like B-cell infiltrates with sparse T-cells, and disrupted CD21 networks, as observed here.^5^^,^^6^ Clinically, pcMZL favors younger patients with trunk and extremity lesions and follows an indolent course, whereas scMZL more often affects older patients, involves the head and neck, and carries greater systemic risk.^5^ A systemic workup, including imaging and bloodwork with flow cytometry is recommended; if initial workup demonstrates concerning findings, follow-up biopsies of lymph nodes, bone marrow, or other suspected involved organs should be considered to distinguish these 2 entities.

Chronic inflammatory states including infections (eg, chronic hepatitis, Epstein-Barr virus, and *Borrelia burgdorferi*) and autoimmune diseases (eg, rheumatoid arthritis, Hashimoto thyroiditis, and Sjogren syndrome) have been associated with pcMZL and other extranodal MZLs.^7^ Chronic antigenic stimulation is a well-recognized driver of MZL pathogenesis. In pcMZL, persistent local inflammation may give rise to a lymphoma clone at the site of stimulation.^1^ In contrast, in our case, the presence of a circulating B-cell population in the blood and bone marrow suggests systemic disease, with the graft site acting as a homing location for malignant cells. Prolonged wound-associated inflammation at the graft site may have provided inflammatory signals and increased blood vessel growth that recruited the aberrant B-cell population to the site.^8^^,^^9^ This mechanism differs from de novo lymphomagenesis in pcMZL but aligns with prior, albeit rare, reports of lymphomas arising preferentially in inflamed or post-surgical tissue due to altered local immune signaling.^4^^,^^10^

Here, we report an unusual case of scMZL development at a prior MMS graft site. While the exact etiology is unknown, this occurrence may be induced by the post-surgical inflammatory state. Despite the rarity of this presentation, clinicians should be aware of the possibility of lymphoma development at surgical sites. Additionally, workup to distinguish between pcMZL and scMZL is critical, as these 2 disease processes have significant differences in morbidity and mortality.

## Conflicts of interest

None disclosed.

## References

[1] Du M.Q. (2016). MALT lymphoma: a paradigm of NF-κB dysregulation. Semin Cancer Biol.

[2] Bradford P.T., Devesa S.S., Anderson W.F., Toro J.R. (2009). Cutaneous lymphoma incidence patterns in the United States: a population-based study of 3884 cases. Blood.

[3] Leary D.O., Goyal N., Rubin N., Goyal A. (2022). Characterization of primary and secondary cutaneous B-Cell lymphomas: a population-based study of 4758 patients. Clin Lymphoma Myeloma Leuk.

[4] dʼAmore E.S.G., Wick M.R., Geisinger K.R., Frizzera G. (1990). Primary malignant lymphoma arising in postmastectomy lymphedema: another facet of the stewart-treves syndrome. Am J Surg Pathol.

[5] Gerami P., Wickless S.C., Querfeld C., Rosen S.T., Kuzel T.M., Guitart J. (2010). Cutaneous involvement with marginal zone lymphoma. J Am Acad Dermatol.

[6] Jaffe E.S. (2020). Navigating the cutaneous B-cell lymphomas: avoiding the rocky shoals. Mod Pathol.

[7] Cerhan J.R., Habermann T.M. (2021). Epidemiology of marginal zone lymphoma. Ann Lymphoma.

[8] Hom D.B. (1994). The wound healing response to grafted tissues. Otolaryngol Clin North Am.

[9] Bain C.R., Myles P.S., Corcoran T., Dieleman J.M. (2023). Postoperative systemic inflammatory dysregulation and corticosteroids: a narrative review. Anaesthesia.

[10] Ramos-Gallardo G., Carballo-Zarate A.A., Cuenca-Pardo J. (2020). What is the evidence of lymphoma in patients with prostheses other than breast implants?. Aesthet Plast Surg.

